# Supplementary figures and images for: Aquorin Bioluminescence-Based Ca2+ Imaging Reveals Differential Calcium Signaling Responses to Abiotic Stresses in Physcomitrella patens
Source: Plants (Basel). 2025 Apr 10;14(8):1178. doi: 10.3390/plants14081178 (PMC12030502; doi:10.3390/plants14081178)

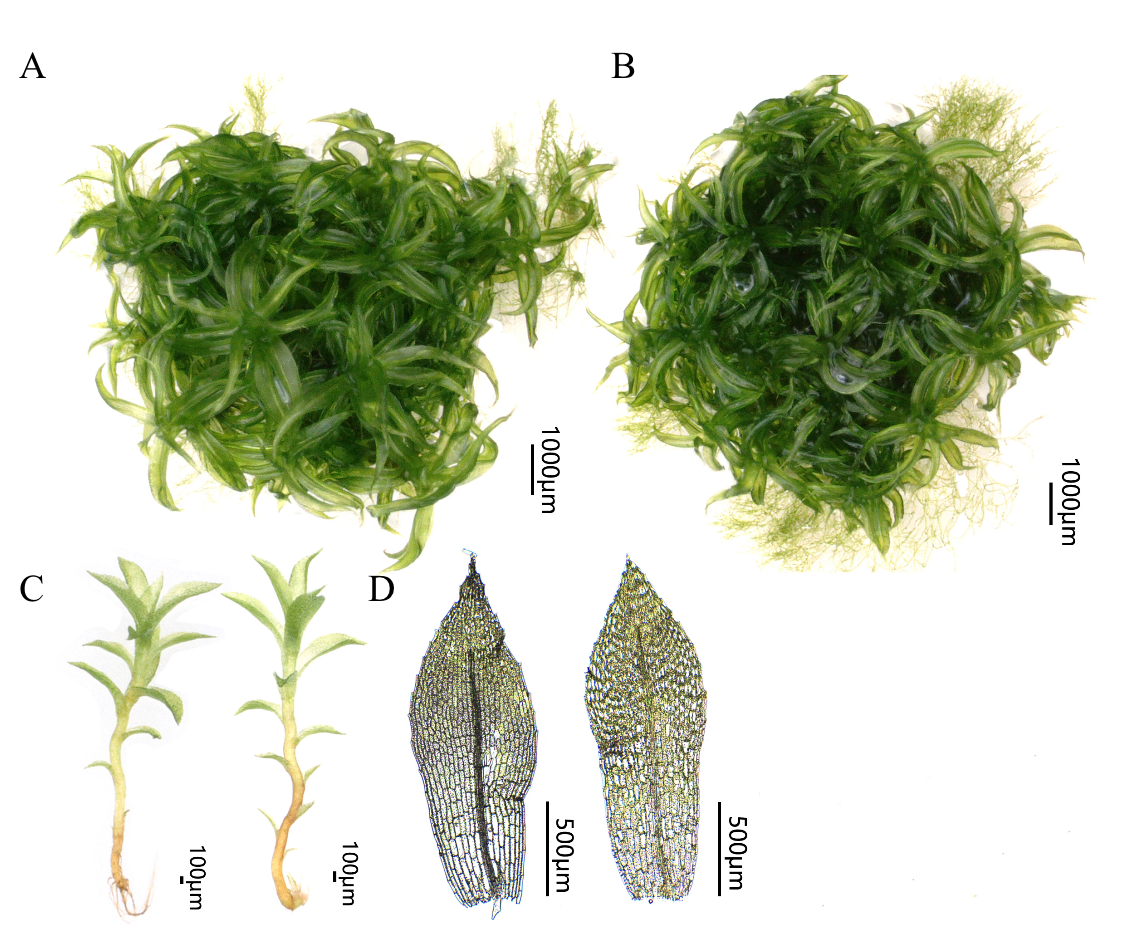

Supplement: Supplementary file 1 [file plants-14-01178-s001.zip › Figure S1.tif]

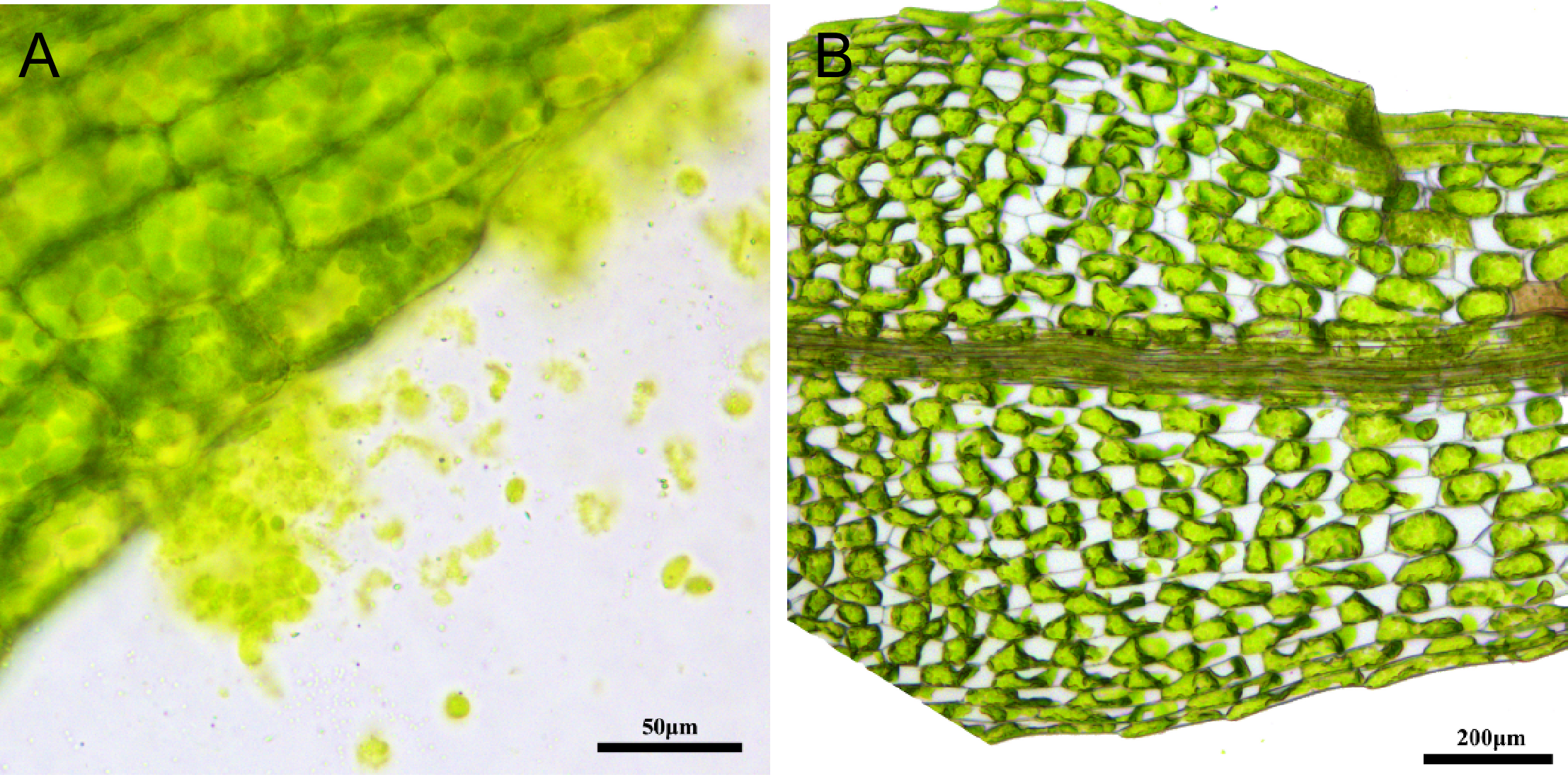

Supplement: Supplementary file 1 [file plants-14-01178-s001.zip › Figure S2.tif]

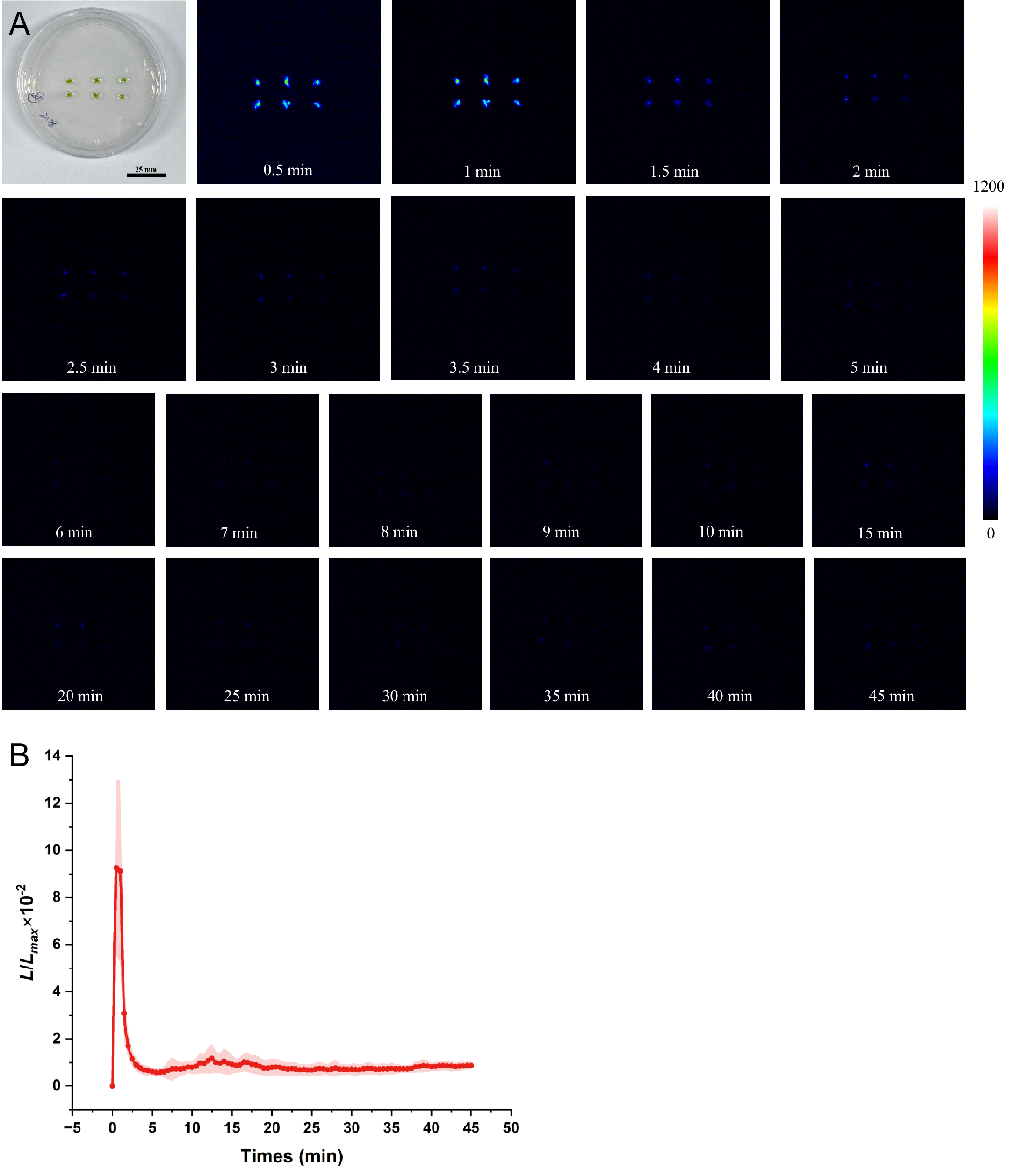

Supplement: Supplementary file 1 [file plants-14-01178-s001.zip › Figure S3.tif]
